# Endothelial Transdifferentiation of Tumor Cells Triggered by the Twist1-Jagged1-KLF4 Axis: Relationship between Cancer Stemness and Angiogenesis

**DOI:** 10.1155/2016/6439864

**Published:** 2015-12-28

**Authors:** Hsiao-Fan Chen, Kou-Juey Wu

**Affiliations:** Research Center for Tumor Medical Science, Graduate Institute of Cancer Biology, China Medical University, Taichung 404, Taiwan

## Abstract

Tumor hypoxia is associated with malignant biological phenotype including enhanced angiogenesis and metastasis. Hypoxia increases the expression of vascular endothelial cell growth factor (VEGF), which directly participates in angiogenesis by recruiting endothelial cells into hypoxic area and stimulating their proliferation, for increasing vascular density. Recent research in tumor biology has focused on the model in which tumor-derived endothelial cells arise from tumor stem-like cells, but the detailed mechanism is not clear. Twist1, an important regulator of epithelial-mesenchymal transition (EMT), has been shown to mediate tumor metastasis and induce tumor angiogenesis. Notch signaling has been demonstrated to be an important player in vascular development and tumor angiogenesis. KLF4 (Krüppel-like factor 4) is a factor commonly used for the generation of induced pluripotent stem (iPS) cells. KLF4 also plays an important role in the differentiation of endothelial cells. Although Twist1 is known as a master regulator of mesoderm development, it is unknown whether Twist1 could be involved in endothelial transdifferentiation of tumor-derived cells. This review focuses on the role of Twist1-Jagged1/Notch-KLF4 axis on tumor-derived endothelial transdifferentiation, tumorigenesis, metastasis, and cancer stemness.

## 1. Introduction

Metastasis and angiogenesis are among the hallmarks of malignant behavior of cancer cells. Cancer metastasis has been shown to be responsible for the majority of cancer-related deaths. It is established that survival rate of cancer patient is low during metastatic stage [1]. Metastasis proceeds through the progressive acquisition of traits that allow malignant cells originating in one organ to disseminate and colonize a secondary site. Metastasis is a multistep process that divides into several steps: loss of cellular adhesion, increased motility and invasiveness, entry and survival in the circulation, exit into new tissue, and eventual colonization in a distant site [2]. A developmental program termed epithelial–mesenchymal transition (EMT) has been shown to play a critical role in promoting metastasis by enhancing cancer cell motility and dissemination. Activation of EMT is considered essential to allow cancer cells to lose cell-cell junctions and dissociate from each other for single-cell migration and invasion [3]. Moreover, gene expression patterns in human cancers indicated that cancer cells combine EMT properties with a stem-cell-like phenotype [4]. A direct molecular link between EMT and stemness has demonstrated that the EMT activator, Twist1, can coinduce EMT and stemness properties [5]. Furthermore, induction of EMT in more-differentiated cancer cells can generate CSC-like cells, providing an association between EMT, CSCs, and drug resistance [6, 7]. Increasing evidence suggests that EMT plays an important role in therapeutic resistance. For example, in* EGFR* mutated non-small cell lung cancers (NSCLC), EMT has been associated with acquired resistance to EGFR inhibitors [8]. EMT also contributes to drug resistance to 5-FU in pancreatic cancer and colon cancer [9, 10]. Due to the clinical importance of the EMT-induced processes, inhibition of EMT is an attractive therapeutic approach that could have a significant effect on disease outcome.

**Figure 1:**
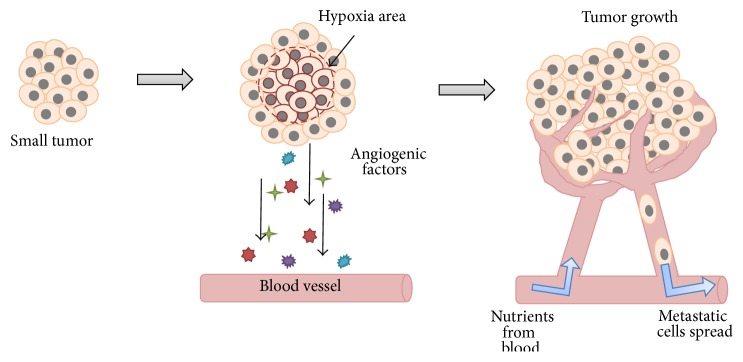
Angiogenesis is the process through which new blood vessels form and grow. Tumor cells activated by a lack of oxygen (or a gene mutation) release, among other things, angiogenic factors that attract inflammatory and endothelial cells and promote their proliferation. The endothelial cells that form existing blood vessels respond to angiogenic signals in their vicinity by proliferating and secreting proteases, which break open the blood vessel wall to enable them to migrate toward the tumor site. Proliferating endothelial cells then organize themselves into new capillary tubes by altering the arrangement of their adherence-membrane proteins. Finally, the capillaries provide a continuous blood flow that sustains tumor cell metabolism and sets up escaping avenues for metastatic tumor cells.

The generation of new capillaries from preexisting blood vessels is called angiogenesis. The angiogenesis process takes place during embryogenesis and in the adult, for example, in the female reproductive system and wound healing. Additional angiogenesis occurs in pathological conditions such as cancer, macular degeneration, psoriasis, and rheumatoid arthritis [11, 12]. Angiogenesis and tumor progression are very closely linked to each other. Tumor cells are dependent on angiogenesis because their growth and expansion require oxygen and nutrients, which are made available through the angiogenic vasculature (Figure 1). In 1971, Folkman proposed that an alteration in the blood supply can noticeably affect the tumor growth and its metastasis, which led to the idea that blocking tumor angiogenesis could be one of the strategies to prevent tumor cells spreading [13–15]. Tumor stem-like cells belong to a subpopulation of tumor cells that have acquired the stemness properties associated with normal stem cells. Cancer stemness property has been used to explain cancer initiation, progression, recurrence, and resistance to chemotherapy or radiation therapy. Recent research in tumor biology has focused on the model in which tumor-derived endothelial cells can arise from tumor stem-like cells [16–18], but the detailed mechanism is not clear. Furthermore, the evidence showed that about 70% of endothelial cells from the inner portion of the tumor were tumor-derived endothelial cells which were stained by human-specific antibody, whereas nearly all the endothelial cells in the tumor capsule were recruited from preexisting vessels which were stained by mouse-specific antibody inside glioblastoma xenografts [16].

Twist1, a basic helix-loop-helix (bHLH) transcription factor, is characterized by a basic DNA binding domain that targets the consensus E-box sequence 5′-CANNTG-3′ [19]. Consistently, bHLH members are transcription factors acting in various differentiation processes, as either positive or negative regulators, and play key roles in different developmental events like neurogenesis and myogenesis [20]. Twist initiates* Drosophila* mesoderm development and results in the formation of heart, somatic muscle, and other cell types [19]. Recent evidence implicates that Twist1 gene is overexpressed in a large of human tumors including a variety of carcinomas as well as sarcomas, melanomas, glioma, and neuroblastoma [21]. Functional studies have indicated that Twist1 may play a major role in tumor promotion and progression, by inhibiting differentiation, interfering with the p53 tumor suppressor pathway and favoring cell survival, and inducing epithelial-mesenchymal transition (EMT) [22].

Here, we discuss the relationship of the EMT regulator, Twist1, cancer stemness, and tumor angiogenesis. We also review the new role of Twist1 in angiogenesis and new downstream targets of Twist1.

## 2. Cancer Stemness and Angiogenesis

Cancer arises from cells accruing multiple mutations which initiate uncontrolled proliferation or resistance to apoptosis by both genetic and epigenetic aberration within unique microenvironments. Moreover, these cells, so-called cancer stem-like cells (CSCs), obtain self-renewing ability as stem-cell-like properties [23]. Some of the pathways activated in CSCs just like in normal stem cells are Notch, Hedgehog, and Wnt/*β*-catenin [24]. They also share similar gene and epigenetic profiles and express related surface and functional markers in different tumors, such as CD44, CD133, ALDH1, Sca1, and ABCG2. Some of these genes or markers also have been proposed for metastasis, angiogenesis, drug resistance, and tissue differentiation [25].

Cancer stem cells are well known for their greater potential of tumor initiation and formation than non-stem tumor cells. Recently, more and more reports support that CSCs, as well their self-renewal and proliferative capabilities, may promote tumor angiogenesis. First, in stem-cell-like glioma cells (SCLGC), Bao et al.'s group observed that the VEGF expression in CD133+ SCLGC was 10–20-fold upregulated, combined with a dramatically increased vascular density identified by CD31 staining [26]. Then, Folkins et al.'s group also revealed that tumor with larger CSC population recruited a higher amount of endothelial progenitor cells (EPC), suggesting that CSCs promote tumor angiogenesis and EPC recruitment via stimulating VEGF and SDF-1 [27]. Recently, the evidences further showed that the presence of cancer-derived endothelial-like cells and suggested that the differentiation of cancer stem-like cells into endothelial cells might be mediated by vascular endothelial growth factor (VEGF) and Notch. These new findings provide new insight into the mechanisms of tumor neoangiogenesis [16, 18]. However, in order to discover the entire network of signals within CSCs and angiogenesis, more research is still needed.

## 3. Hypoxia-Induced EMT

Hypoxia is an important physiological factor that correlates with tumor progression including an increasing probability of recurrence, locoregional spread, and distant metastasis [28]. Furthermore, recent studies suggest that tumor hypoxia is associated with malignant biological phenotype such as angiogenesis, migration, invasion, and metastasis [29]. The key factor involved in adaptive responses to cellular hypoxia is HIF-1 and its activity is tightly regulated by the cellular oxygen tension [30]. HIF-1 is a heterodimeric protein that is composed of an O2-regulated HIF-1alpha subunit and a constitutively expressed HIF-1beta subunit. Both of them belong to the basic helix-loop-helix-per-arnt-sim (bHLH-PAS) family [31]. Hypoxia mediates EMT and metastasis. Twist1 is a direct gene target of HIF-1alpha and Twist1 mediates the invasion, migration, and metastatic activity of different cancer cell types, including head and neck (HNSCC), breast, and lung carcinoma [32].

## 4. Hypoxia-Induced Tumor Angiogenesis

Typically, tumor-associated angiogenesis goes through two phases: an avascular and a vascular phase that are separated by the “angiogenic switch.” In the avascular phase, tumors are small and survive on diffusion of nutrients from the host microvasculature. In order for tumors to grow beyond 1-2 *μ*m^3^ [33], they need a continual supply of blood to supply nutrients and oxygen to overcome hypoxia and starvation. Hypoxia of tumor cells will occur if the tumor grows beyond the maximum distance of diffusion from local vessels around 200 *μ*m [34]. When a condition such as hypoxia is present in the tumor tissue, the tumor cells receive the signal and promote the angiogenic switch and induce angiogenesis. In the case of hypoxia, the signal is mediated by hypoxia inducible factor-1 (HIF-1). HIF-1 binds to hypoxia-response elements (HREs) and activates a number of hypoxia-response genes such as VEGF. Thus hypoxia upregulates the expression of angiogenic factors, like VEGF, stromal derived factor 1 (SDF1), angiopoietin 2 (ANGPT2), placental growth factor (PGF), platelet-derived growth factor B (PDGFB), and stem cell factor (SCF) [35–40]. Receptor-ligand interaction activates these cells and promotes the recruiting endothelial cells into hypoxic area and stimulates their proliferation, for increasing vascular density [41].

## 5. Role of Twist1 in EMT and Angiogenesis

The mechanisms leading to the aberrant activation of Twist1 appear to be various and complex. They result from the deregulation of signaling pathways (e.g., transforming growth factor-beta (TGF-*β*), Wnt, and nuclear factor *κ*B (NF-*κ*B) signaling pathways) that normally mediate the expression of the genes during embryonic development [42]. Interestingly, stress conditions seem to control both the physiological and aberrant expression of Twist1. Hypoxic conditions are similarly defined as potent inducers of Twist1 expression in cancer cells, thereby promoting cell dissemination to other friendlier environment, presumably through its role in promoting the EMT and metastasis [32]. Besides EMT and metastasis, the recent finding provides a crucial link between less differentiated stem cells and the mesenchymal-appearing cells generated by EMTs [43]. Our results demonstrated that Twist-induced EMT and tumor-initiating capability in cancer cells occur through direct regulation of the polycomb group protein BMI1, which is involved in the self-renewal of neuronal, haematopoietic, and intestinal cells [5]. In addition, it was found that upregulation of Twist1 may play an important role in the angiogenesis of breast and hepatocellular carcinoma [44, 45]. But so far, the molecular mechanism of Twist1 gene on angiogenesis in human cancers remains unknown. The identification of downstream activators of Twist1 could provide valuable information about tumor angiogenesis and metastasis.

## 6. The Role of Notch Signaling Pathway in EMT and Angiogenesis

The Notch-signaling pathway is a cell-cell communication pathway that is evolutionarily conserved from* Drosophila* to human and modulates cell fate and differentiation [46–48]. To date, four different notch receptors (Notch1, Notch2, Notch3, and Notch4) and five different ligands (Jagged1 and Jagged2 and Delta-like-1, Delta-like-3, and Delta-like-4) have been identified in mammalian cells. Notch signaling is initiated when the extracellular domain of the Notch receptor binds their ligand on neighboring cells that are in close proximity to one another. This leads to a cascade of enzymatic cleavages and the Notch intracellular domain (NICD) is released and then translocated to the nucleus where it interacts with CSL (CBF1, Su(H), and Lag-2) transcriptional repressors and converts them to transcriptional activators.

Recently, it is believed that Notch signal pathway is a key regulator to induce EMT and endothelial-to-mesenchymal transition (EndMT) processes [49, 50]. Notch activation in endothelial cells results in morphological, phenotypic, and functional changes consistent with mesenchymal transformation. These changes not only include downregulation of endothelial markers (VE-cadherin, Tie1, Tie2, platelet-endothelial cell adhesion molecule-1, and endothelial NO synthase), but also upregulation of mesenchymal markers (*α*-SMA, fibronectin, and platelet-derived growth factor receptors) [51]. Moreover, Jagged1 stimulation in endothelial cells also induced a similar mesenchymal transformation, suggesting that Jagged1 mediated activation of Notch signaling is important during the induction of EMT [51]. In EndMT and EMT processes, Notch cross-talks with several transcription and growth factors relevant to EMT, including Snail, Slug, TGF-*β*, FGF, and PDGF [52].

It is clear that the Notch family is critically important for the proper construction of the vascular system. Global as well as endothelium-specific knockouts of Notch receptors or ligands induce embryonic death with vascular defects [53–55]. These results suggest that Notch pathway components have also been shown to be required for postnatal angiogenesis. However, information about Notch signaling in tumor angiogenesis is limited. Notch signaling components are expressed in tumor endothelial cells, but the most notable component in this class is DLL4. It is known that DLL4 is upregulated in the vasculature of human xenografted tumors in mice and in human breast and kidney cancers [56]. Reduction of basal DLL4 level in ECs by siRNA led to the inhibition of multiple endothelial functions* in vitro* including proliferation, migration, and network formation, implying the potential role of this pathway in cancer [57]. In fact, blockade of DLL4-Notch signaling is an emerging therapeutic approach to inhibiting tumor angiogenesis [58–60]. Besides, recent findings suggest that the role of Jagged1 expression in head and neck squamous cell carcinoma and breast cancer can be diverse, influencing tumor cell growth, tumor angiogenesis, and/or the inflammatory response [61, 62].

## 7. The Role of KLF4 in EMT and Angiogenesis

KLF4 is member of the Sp1/KLF family, which are evolutionarily conserved zinc finger-containing transcription factors and function as regulators in diverse cell processes of cell growth, proliferation, and differentiation [63–65]. Earlier studies indicated that KLF4 is highly expressed in epithelial tissues including the gut and skin [66, 67]. Because KLF4 functions as an antiproliferative factor in differentiated epithelia, it seems that KLF4 might act as a tumor suppressor. In general, KLF4 seems to inhibit both EMT and invasion [68]. While loss of KLF4 function induces EMT-like morphological changes, forced expression of KLF4 in the highly metastatic MDA-MB-231 breast tumor cell line was sufficient to restore E-cadherin expression and suppress migration and invasion [69]. Furthermore, NFI-C, a member of the nuclear factor I (NFI) family of transcription factors, increased the expression of KLF4 and E-cadherin and led to a more pronounced epithelial cell phenotype. In contrast, NFI-C knockdown induced migration and invasion [70]. Notably, the research revealed that a number of mesenchymal genes, such as N-cadherin (Cdh2), vimentin (Vim), and *β*-catenin (Ctnnb1), are direct targets of KLF4 transcriptional repression by using a combinatorial approach of gene expression profiling and chromatin immunoprecipitation/deep sequencing (ChIP-Seq) analysis [71]. KLF4 significantly decreases lung and liver metastases in a murine model of mammary cancer [69, 72]. Indeed, loss of KLF4 occurs at early stages in the progression of gastric cancer [73, 74]. However, recent evidence suggests that KLF4 might also act as an oncogene in breast cancer, head and neck cancer (HNSCC), and pancreatic cancer [75–78]. It indicated that KLF4 expression and activity are altered in human cancers and KLF4 can be tumor suppressors or oncogenes depending on tissue, tumor type, or cancer stage.

It was found that overexpression of KLF4 along with Myc, Sox2, and Oct4 could transform mouse fibroblasts into the state resembling embryonic stem cells (ES cells). These cells have been termed “inducible pluripotent stem cells” (iPS cells) [79]. There are also some studies implying that KLF4 played an important role in the differentiation and function of endothelial and vascular smooth muscle cells [59, 80–82]. Furthermore, it is demonstrated that KLF4 can regulate sprouting angiogenesis and may be a therapeutic target in regulation of tumor angiogenesis [83].

## 8. Twist1 Induced Tumor-Derived Endothelial Differentiation

There are some evidences that glioblastoma stem-like cells differentiate into endothelial cells [16, 17], but the detailed molecular mechanisms are still unclear. We demonstrated that Twist1 overexpression in the HNSCC cell lines not only mediates the expression of the endothelial-specific markers including CD31 [84], CD144 [85], von Willebrand factor (vWF) [86], Tie2 [87], endoglin (CD105) [88], and intercellular adhesion molecule 1 (ICAM1) [89], but also exhibited obvious ability of capillary-like network formation and the ability of DiI-AcLDL (1,1′-dioctadecyl-3,3,3′,3′-tetramethyl-indocarbocyanide perchlorate-labeled acetylated low density lipoproteins) uptake [90, 91]. It is a new vision that Twist1 can induce transdifferentiation of tumor cells into endothelial cells and promotion of tumor-derived vascular formation [18]. This observation of tumor-derived endothelial transdifferentiation is different from the traditional angiogenesis process contributed by sprouting and proliferation of formerly quiescent endothelial cells on nearby blood vessels and lymphatics that are triggered by soluble growth factors, cytokines, and proangiogenic factors secreted from tumor cell (Figure 1) [92]. Induction of tumor-derived endothelial differentiation by Twist1 was also different from the vasculogenic mimicry mechanism [93], because vasculogenic mimicry is the process by which aggressive tumor cells generate nonendothelial cell-lined channels delimited by extracellular matrix. Knockdown of Twist1 expression decreased not only cell mobility but also the tube-forming ability. Tumor-derived endothelial differentiation is important for Twist1-induced tumor metastasis, and inhibition of the angiogenesis process may be equally important to treat metastasis [18]. Finally, how classical angiogenesis versus endothelial transdifferentiation contributes to tumor angiogenesis and whether these two different mechanisms occur sequentially or have any tumor type preference remain to be determined through examination of different types of human tumors.

## 9. Regulation of the Jagged1-KLF4 Axis by Twist1

Recent study showed that the Twist1 functions upstream of Jagged1 in the process of development [94], but the regulatory mechanism was not provided. Our results indicate that Twist1 can activate Jagged1 expression and downstream Notch signaling pathway. In addition, the reporter assay and chromatin immunoprecipitation (ChIP) assay were performed and confirmed that Twist1 activated the expression of Jagged1 by directly binding to the E-box element in the Jagged1 promoter. Knockdown of Jagged1 not only decreased the levels of endothelial markers including CD31, CD144, vWF, CD105, and ICAM1 induced by Twist1 overexpression, but also abolished the activity of tube formation and DiI-AcLDL uptake activity induced by Twist1. Then, downregulation of Jagged1 caused the reverse shift in expression of mesenchymal markers (vimentin and N-cadherin) to epithelial markers (E-cadherin and plakoglobin) and abolished Twist1-mediated migration/invasion activity. Taken together, these results demonstrated that Jagged1 plays an essential role in Twist1-induced endothelial differentiation, EMT, and metastasis. Furthermore, the relationship among Notch, STAT3, and Twist1 pathways in the control of tumor progression was studied, and the results suggested that Notch1/STAT3/Twist signaling axis is involved in progression of human gastric cancer [95]. It provides an idea that there might be a positive feedback loop between Twist pathway and Notch signaling to promote tumor progression.

As Twist1 overexpression was shown to generate cells with stem-like properties [5], there are more and more evidences showing that Notch signaling pathway is involved in adult stem cell self-renewal and differentiation [96–98]. Moreover, recent researches indicated that tumor stem-like cell differentiation to endothelial-cell progenitors occurs trough Notch-mediated signaling [99]. Some pluripotency factors had an essential function in this network by actively directing differentiation for endoderm specification [100]. To further identify the transcription factors as downstream targets of the Twist1-Jagged1/Notch signaling to regulate the expression of various endothelial and vascular markers, we screened the expression of different stemness-related transcriptional factors including OCT4, SOX2, NANOG, KLF4, GFI1, WNT1, and BMI1. The results showed that Jagged1/Notch pathway can regulate the expression of KLF4 by directly binding to the KLF4 promoter using the qChIP assay. Although KLF4 is very likely an important regulator of ES cell self-renewal and pluripotency, our results demonstrate a role of KLF4 in endothelial differentiation and vasculogenesis. The direct regulation of KLF4 also showed the connection between the Notch pathway and KLF4. The potential downstream targets of KLF4 (e.g., Wnt5A, CCND2) may give us a new thought in the mechanism of KLF4-induced stem-like property that contributes to the tumor-initiating ability. Finally, KLF4 mediates Twist1-induced metastatic activity through an EMT-independent mechanism, suggesting that regulation of different targets (e.g., motility genes) other than the typical EMT marker genes also contributes to the metastatic activity induced by Twist1. All these results indicate the role of KLF4 in Twist1-induced endothelial differentiation, stem-like property, and metastasis.

## 10. Clinical Impaction of Twist1-Jagged1/KLF4 Axis

Furthermore, we also examined the correlation between the expression of Twist1, Jagged1, and KLF4 in head and neck cancer patient samples. Immunohistochemistry staining of Twist1, Jagged1, and KLF4 in 242 head and neck cancer patient samples showed there was significant correlation between Twist1, Jagged1, and KLF4. Meanwhile, the expression of Twist1-Jagged1-KLF4 axis was also confirmed in primary culture samples derived from head and neck samples. Overall, these results indicated that Twist1-Jagged1-KLF4 axis existed in real patient samples.

Cetuximab was recently approved in combination treatment with cisplatin for the treatment of patients with squamous cell carcinoma of the head and neck, but the survival benefit of adding cetuximab to standard chemotherapy was almost only three months [101, 102]. This means that there is still room for further improvement of treatment approach to treating head and neck cancer. It is well established that the angiogenic switch is a critical step in carcinogenesis [103]. With the clinical application of multiple inhibitors of vascular endothelial growth factor (VEGF) signaling, angiogenesis is a validated therapeutic target [13, 92]. However, the overall clinical benefit of agents targeting VEGF has been less than what was hoped. This lack of benefit appears to be substantially due to primary or acquired resistance to these drugs [104]. The tumor-derived endothelial differentiation might be responsible for this resistance. Because the Twist1-Jagged1-KLF4 axis seems to play an important role in angiogenesis, blocking Notch signaling activation by *γ*-secretase inhibitors might be a potential treatment.

Over the past decades, *γ*-secretase inhibitors have been investigated for their clinical potential to block the generation of A*β* peptide that is associated with Alzheimer's disease [105]. Because *γ*-secretase inhibitors are also able to prevent Notch receptor activation, several forms of *γ*-secretase inhibitors have been tested for cancer therapy. Treatment with one of *γ*-secretase inhibitors, N-[N-(3,5-difluorophenacetyl)-L-alanyl]-S-phenylglycine t-butyl ester (DAPT), either reduced medulloblastoma growth in a SmoA1 mouse model or induced G0-G1 cell cycle arrest and apoptosis in a T-ALL mouse model [106, 107]. Furthermore, a Notch inhibitor, MK0752, has been used for T-ALL patients and advanced breast cancers for a phase I clinical trial [108, 109]. To investigate whether the existence of Twist1-Jagged1-KLF4 axis might provide a potential new strategy treatment for the patients with Twist1-overexpressing tumors, we tested the drug response on Twist1-overexpressing OECM-1 cells. Xenotransplantation experiments showed that combined treatment of cetuximab and DAPT additively inhibited the tumor growth induced by Twist1 [18]. These results indicate the benefit of the *γ*-secretase inhibitor (DAPT) in combination treatment for Twist1-overexpressing tumors. However, further development of a specific type of *γ*-secretase inhibitor that can specifically inhibit certain human tumors needs to be initiated in order to guarantee the success of target therapy of human cancers.

## 11. Conclusion

**Figure 2:**
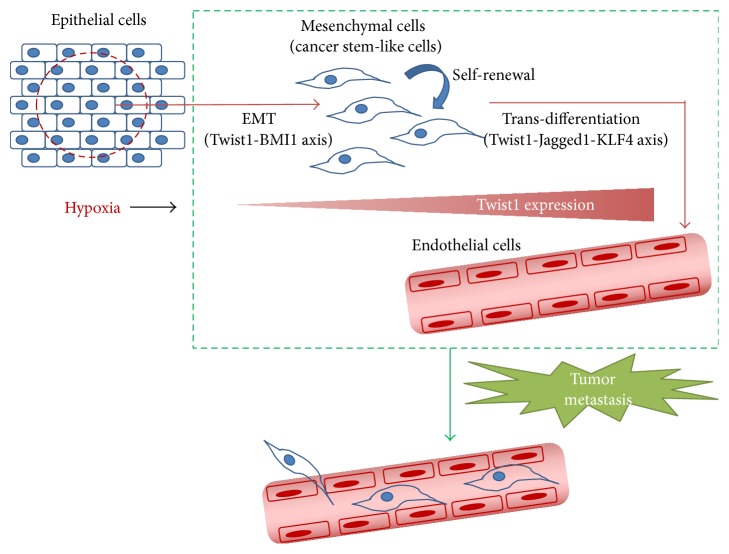
A model explains the crucial role of hypoxia-induced Twist1 to mediate different important processes of tumor progression including EMT, metastasis, cancer stemness, and endothelial differentiation through regulation of BMI1 or Jagged1/Notch-KLF4 axis.

Tumor hypoxia is associated with malignant biological phenotype including enhanced invasiveness, angiogenesis, migration, and metastasis. HIF-1alpha, a key transcription factor that is induced by hypoxia and is implicated in tumor progression/metastasis, induces EMT through direct activation of Twist1 [32]. Twist1 plays a crucial role in epithelial-mesenchymal transition (EMT), metastasis, and cancer stemness through direct regulation of BMI1 [5]. Cancer stem cells have been described to be critical in tumor initiation tumor growth and metastasis. More evidences have shown that CSCs interact closely with angiogenesis and have the potential to develop the blood vessels [99]. Furthermore, our results indicate that the Twist1-Jagged1-KLF4 axis plays an important and essential role in inducing tumor-derived endothelial differentiation inside the tumors in addition to traditional angiogenesis and in creating better opportunities for tumor metastasis (Figure 2). These results also provide significant therapeutic implications to combine *γ*-secretase inhibitors with established chemotherapeutic agents for cancer treatment.
